# The Observer

**Published:** 1896-10

**Authors:** 


**The Observer.** An illustrated monthly magazine of the Outdoor World and Microscopy. E. P. Bigelow, publisher, Portland, Conn. Subscription price, $1.00 a year. 10 cents a number.

The August and September numbers of this elegant magazine are before us. The journal has been noticed in these pages before.

Among the articles we notice the following: "A Day's Outing at Santa Monica," by F. E. Gray. "A What Is It?" by G. E. Davis. This is an interesting article on the ant-lion. "Ferns and Fern Lore," by W. N. Cute, is another fine article. This article is beautifully illustrated. "Three Hours in the Caverns of Luray," by Amadeus W. Grabau, is an interesting statement concerning one of the most wonderful as well as magnificent caves in the world. "Some River Phenomena," by F. P. Gorham, gives a description of remarkable effects in the Blackstone River. "Critical Periods in the History of the Earth," by Arthur M. Edwards, M. D., is another good article. Mr. J. E. Huber writes about bullfrogs. A number of articles in the department of Ornithology, and several in the department of Astronomy, are of exceptional interest. The Microscopical department is beautifully illustrated. New England Orchids will interest those who make a specialty of botany. "First Steps in the Study of Fishes," by Dr. R. W. Shufeldt, will help all who wish to get an orderly knowledge of fishes, in other words, a classified knowledge of fishes. Observations upon "The Trap-door Spider" is another contribution.

The above list of articles shows the scope of the journal, and all are of reasonable length and not of such technical style as to render them difficult to read.

